# X-ray phase contrast tomography by tracking near field speckle

**DOI:** 10.1038/srep08762

**Published:** 2015-03-04

**Authors:** Hongchang Wang, Sebastien Berujon, Julia Herzen, Robert Atwood, David Laundy, Alexander Hipp, Kawal Sawhney

**Affiliations:** 1 Diamond Light Source Ltd, Harwell Science and Innovation Campus, Didcot, OX11 0DE, UK; 2 Institue of Materials Science, Helmholtz-Zentrum Geesthacht, Geesthacht, Germany

## Abstract

X-ray imaging techniques that capture variations in the x-ray phase can yield higher contrast images with lower x-ray dose than is possible with conventional absorption radiography. However, the extraction of phase information is often more difficult than the extraction of absorption information and requires a more sophisticated experimental arrangement. We here report a method for three-dimensional (3D) X-ray phase contrast computed tomography (CT) which gives quantitative volumetric information on the real part of the refractive index. The method is based on the recently developed X-ray speckle tracking technique in which the displacement of near field speckle is tracked using a digital image correlation algorithm. In addition to differential phase contrast projection images, the method allows the dark-field images to be simultaneously extracted. After reconstruction, compared to conventional absorption CT images, the 3D phase CT images show greatly enhanced contrast. This new imaging method has advantages compared to other X-ray imaging methods in simplicity of experimental arrangement, speed of measurement and relative insensitivity to beam movements. These features make the technique an attractive candidate for material imaging such as *in-vivo* imaging of biological systems containing soft tissue.

X-rays have been used for imaging since their discovery in the late 19^th^ century as hard X-rays are sufficiently penetrating to pass through biological tissue. The simplest X-ray imaging technique is radiography, where a beam of X-rays pass through a sample and the transmitted intensity is recorded on an area detector. Computed tomography (CT) scanning allows reconstruction of the X-ray absorption as a function of position along the beam path to give a three-dimensional (3D) map of the absorption in the sample. CT scanning using absorption radiography is widely used as a medical diagnostic tool. Absorption contrast is however weak, if the elemental composition and density do not vary significantly within the specimen. This lack of sensitivity makes imaging of biological structures, such as blood vessels in soft tissues, difficult when using absorption-based imaging. Sample dose in absorption based imaging is also a major problem for biological systems, as radiation damage causes structural changes.

The propagation of X-rays depends on the complex X-ray refractive index *n =* 1*-δ-iβ* where the imaginary part (*iβ*) gives rise to X-ray absorption and the real part decrement (*δ*) gives rise to a phase shift. The absorption term is responsible for the contrast observed in absorption radiography and also for radiation dose in the sample. Conversely, the phase shift term generates contrast in phase sensitive radiography but does not cause any sample radiation dose. In addition, spatial variation of the electron density causes scattering by diffraction which results in contrast in the dark-field image. This scattering is stronger when the gradient of the electron density is higher and hence the dark-field image is most sensitive to small structures within the sample.

In the hard X-ray region at X-ray energies away from absorption edges, the absorption term *δ* falls more rapidly with X-ray energy than the phase shift term i*β*. Above 10 keV, for biological samples composed of light elements (carbon, oxygen, hydrogen etc.), *δ* is typically three orders of magnitude larger than *β* and therefore phase sensitive imaging can allow high contrast with lower radiation dose than conventional absorption radiography. Phase contrast techniques have become more widespread with the availability of intense synchrotron radiation X-ray sources and more efficient X-ray detectors with sub-micron spatial resolution^1^,^2^,^3^,^4^,^5^,^6^,^7^. Additionally, dark-field imaging can provide information on the structure of the objects at a scale where the scattering features are smaller than the spatial resolution of the technique^8^,^9^,^10^,^11^,^12^,^13^. Dark-field imaging was first introduced in electron microscopy in the late 1930s and it has taken a further 70 years for it to be realized with X-ray light, mainly due to the lack of efficient X-ray optics^8^,^9^,^10^.

Although several X-ray phase contrast imaging techniques have been developed in the past two decades that are capable of imaging soft tissue, only analyzer-based and grating-based methods are commonly used to obtain both phase and dark-field contrast^3^,^7^,^11^,^14^,^15^,^16^. The analyzer based technique uses a Bragg reflecting perfect single crystal with low angular acceptance in order to measure deflection of the wavefront, however detected intensity is low resulting in a poor signal to noise ratio, the stability requirements are stringent and the technique is sensitive to deflections in only one direction. The grating based technique has been increasing in popularity, especially since its adaptation to low brilliance X-ray tube sources^11^,^17^. The Moiré fringe analysis mode only requires a single fringe image^18^,^19^, but the resolution becomes limited by the fringe or grid spacing when working with 2D interferometers^20^,^21^. To increase the spatial resolution the measurements are frequently done in phase-stepping mode where one grating is scanned relative to the other, transversely to the incident beam^16^. This, however, results in an increased radiation dose in the sample.

In this letter, we describe a new approach for X-ray phase contrast tomography based on the tracking of X-ray near-field speckle, a technique that was originally developed for two-dimensional (2D) X-ray wavefront sensing and imaging^22^,^23^,^24^. We demonstrate extension of the X-ray speckle tracking (XST) method from 2D projection to give 3D volumetric phase information using CT reconstruction and we also show how the approach can be extended to the simultaneous retrieval of dark-field images. To demonstrate the capabilities of the XST CT technique and its potential application to biomedical research, we present data collected on an *ex vivo* human carotid artery specimen^25^,^26^.

The experimental set-up is relatively simple involving only the specimen, an X-ray imaging detector and a phase object containing random high spatial frequency structures, for example a biological filtering membrane or a sheet of abrasive paper. The choice of material and structure size of the phase object depends on the X-ray energy, the setup geometry and the detector resolution. The experimental layout is shown in Fig. 1. In Fig. 1a, a partially coherent X-ray beam passes through the abrasive paper and a speckle pattern is produced on the detector caused by interference of the randomly scattered radiation. One image is firstly taken of the speckle and then a second image is taken with the specimen inserted (Fig. 1b). Fig. 1c and Fig. 1d show a small subset of the two speckle images. In order to track the changes to the speckle between the two images, a digital image correlation algorithm (DIC) is employed^27^. DIC algorithms have been widely employed for deformation of surface mapping where tracked displacement has been shown to be measurable with sub-pixel accuracy. In our application of the correlation algorithm, the subsets were normalized to increase robustness to illumination variation with time. The speckle intensity for a subset with points (*x*_i_, *y*_i_) in a target image and (*x*_i_, *y*_i_) of a reference image is denoted by the functions *f*(*x*_i_, *y*_i_) and 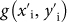, respectively^28^. For a target subset centred in pixel (*x_0_*, *y*_0_), the normalized correlation coefficient *r*_i,j_ is defined as
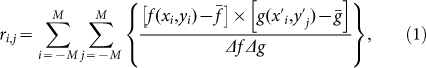
where the number of points along *x* or *y* is 2*M* + 1, and 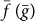 and Δ*f* (Δ*g*) is the mean and standard deviation of the subset, respectively.

The relation between the position of the region without sample (*x*_i_, *y*_i_) and with sample 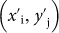 can be written
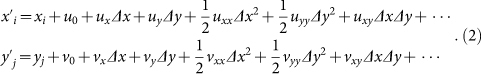


The zero order term (*u*_0_, *v*_0_) describes a rigid body translation of the subset with no change in the speckle pattern. The shape of the reference region can however change between the two images: whilst the first-order term models linear deformations (rotations, shears etc.) of the subset, higher order terms are sometimes used to model more complicated non linear deformations. Scattering from the sample due to strong and rapid variations in the electronic density decreases the beam coherence and deforms locally the speckle pattern. Since the higher orders shape functions account for deformations due to strong and rapid variations in the electronic density, they also mirror the sample scattering. This translates in a reduction in the correlation value between the reference and target subsets. This approach is fully equivalent in principle to scattering properties mapping^29^, where the correlation amplitude and tracking is calculated from a regular grid pattern rather than here a random speckle pattern.

Using equation (1), a correlation map can be generated for every subset of each reference-sample image pair. Fig. 1e shows the correlation map obtained from the reference subset (Fig. 1c) and target subset (Fig. 1d). The peak value *r*_i,j_ and its position (*u*_i,j_, *v*_i,j_) in the correlation map is calculated for each subset of each image. With *u*_i,j_ and *v*_i,j_ corresponding to the in-plane local displacement vector of the speckle pattern caused by refraction in the sample, two displacement maps (*u*, *v*) can be made as shown in Fig. 1f and Fig. 1g. The wavefront gradient is calculated as the ratio of this rigid body translation to the distance between the membrane and detector. The phase shift is then derived from the two transverse phase gradients^30^. The corresponding correlation coefficient map (*r*) is shown in Fig. 1h. The correlation coefficient *r* is equal to unity if the subsets are related by a simple translation and is smaller when the target speckle pattern is distorted due to scattering. The correlation coefficient is related therefore to the scattering from the sample and can be used to generate a dark-field image.

## Results

**Figure 1:**
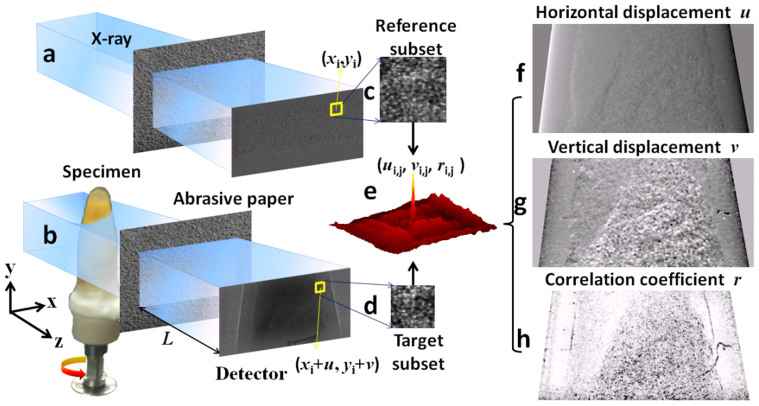
Schematic representation of the experiment setup. Speckle image (a) without the specimen and (b) with the specimen and the phase object (abrasive paper), (c) reference and (d) target subset speckle image, (e) example of correlation coefficient map for the subset, (f) horizontal and (g) vertical displacement and (h) correlation coefficient image.

**Figure 2:**
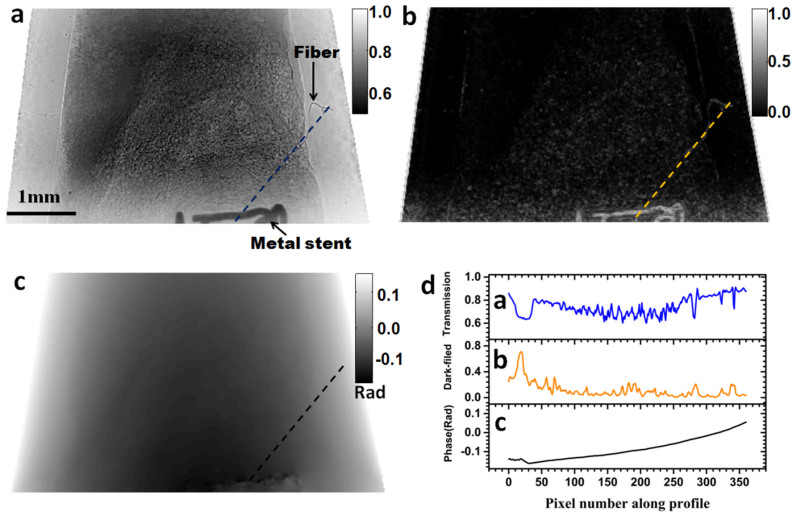
Transmission, dark-field and projected phase image for a human carotid artery. (a) Transmission image, (b) dark-field image, (c) projected phase image and (d) section profiles across the corresponding images of the artery sample (indicated by the coloured line in each image).

Fig. 2 shows one of the projection images obtained using the XST technique applied to a human carotid artery sample. The field of view was 3.4 mm vertically by 6.2 mm horizontally. An X-ray transmission image is shown in Fig. 2a, and Fig. 2b and Fig. 2c show images of the dark-field and phase signals respectively. The X-ray phase gradients in the horizontal (*x*) and vertical (*y*) directions were calculated from the speckle displacement (*u*, *v*) of Fig. 1f and Fig. 1g^22^. The phase shift induced by the artery sample shown in Fig. 2b was reconstructed using the two transverse phase gradients as an input into a pseudo-inversion matrix algorithm. Since the correlation coefficient reduces as the scattering power increases, the dark-field image (*df*) of Fig. 2b was calculated by subtraction of the correlation coefficient map (Fig. 1h) from unity with *df* = 1-*r*.

In Fig. 2a, the metal stent together with one identified fibre outside of the plastic Eppendorf tube are clearly visible in the conventional radiograph. It should be noted that the transmission at the bottom of the image is slightly higher than the rest of the image. This was caused by detector background which could be improved with a dark field correction to the data. As shown in Fig. 2a, the presence of small features in this image is observable due to propagation-based phase contrast edge enhancement. In contrast, the dark-field contrast image (Fig. 2b) yields stronger contrast in regions with more inhomogeneous material and at the feature edges since they scatter more. Thus, scattering information from fibre and the metal stent are clearly visible in the dark-field image. The corresponding projected phase image (Fig. 2c) shows that the phase change is dominated by the Eppendorf tube rather than the sample. As we shall see further, upon CT reconstruction, the sample phase change becomes clearly distinguishable from the container phase shift and the differences in sample density can be best visualized from the reconstructed CT image. Fig. 2(d) shows line profiles marked by the dashed lines in the transmission, dark-field and the phase images. As a comment, one can note that the scattering from the metal stent is much stronger than from the sample even though the transmission signals are similar to each other. Hence, the dark-field image reveals complementary information to the transmission and phase images.

**Figure 3:**
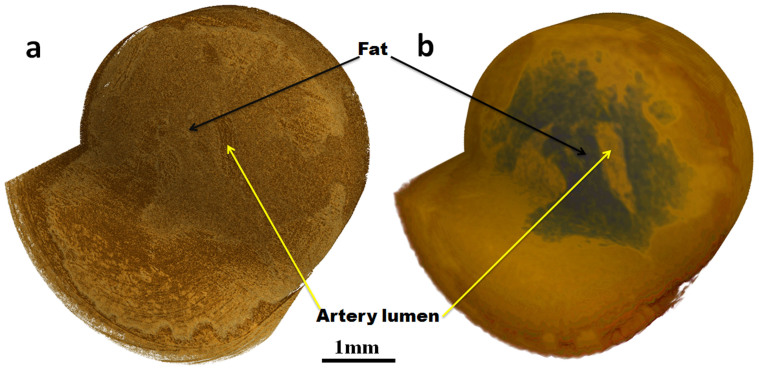
3D rendering of the tomographic reconstruction. Volume rendering from (a) transmission and (b) phase slices.

CT reconstructions were performed using both the transmission and phase image sets. The acquisitions were obtained with the sample mounted on a vertical rotation axis. A set of 900 projections with orientation angles ranging from 0° to 180° were used. Phase signal for all the speckle images were calculated on a parallel processing Central Processing Unit (CPU) computation cluster and later the volume reconstruction was done on a Graphics Processing Unit (GPU) cluster. Fig. 3a and Fig. 3b show the transmission and phase reconstructions of the artery specimen. The volume reconstruction from transmission projection reveals very fine structure in its rendering (mostly in fatty tissue), contrary to the reconstruction from the phase image which reveals more clearly the artery vessel.

**Figure 4:**
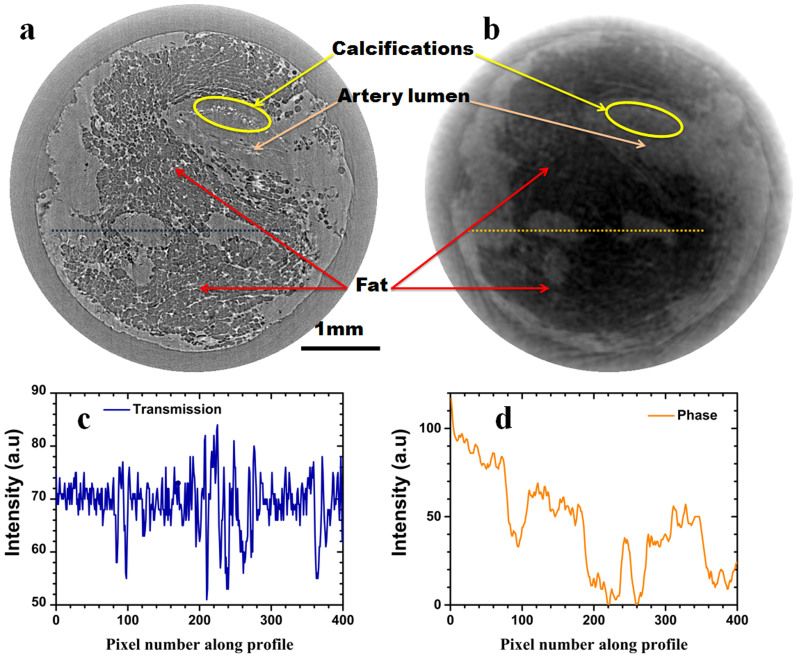
Reconstructed axial slices of the artery specimen. (a) Transmission and (b) phase axial slice, (c) and (d) are line profiles of the image greyscale (8 bit) at the positions indicated by the thin lines from (a) and (b).

Axial axis slices of the displayed reconstructions in Fig. 3 are shown in Fig. 4. Such a view provides an easier way to compare the phase (Fig. 4b) and absorption (Fig. 4a) information. On one hand, the transmission signal shows strong edge enhancement from interference due to the high coherence of the X-ray beam. One can observe in this slice the fatty tissue, small features showing a high level of detail. This is illustrated by the micro calcifications present in the vessel wall circled in yellow in Fig. 4. On the other hand, the phase slice shows more contrast between the artery lumen and fat, detail that is less visible in the transmission image.

Line profiles for the absorption and phase slices are highlighted in Fig. 4c and Fig. 4d. Again, spatial high frequency features are less visible in the phase slice compared to the absorption slice that benefits from the edge enhancement effect. Nevertheless, there is a clear distinction in contrast between sample materials; the line profiles in Fig. 4 clearly show a consistently higher brightness of the lumen over the fat material in the phase image (Fig. 4d) whereas the brightness is much closer in the absorption image (Fig. 4c). The lower spatial resolution of the phase slice is exacerbated by the requirement that several pixels be used to track the speckle displacement with the digital correlation algorithm^22^.

## Summary and Discussion

In summary, the speckle tracking technique has been used to generate multiple phase contrast and dark-field images of a human carotid artery sample at different projection angles. Computed tomography reconstruction was then performed to image a specimen containing a human carotid artery. With a simple experimental arrangement, the speckle based technique is easily implementable at a synchrotron radiation beamline since no perfect crystals analysers or precision gratings are required. Moreover, the speckle based technique causes low sample dosage relative to other differential phase contrast imaging techniques as only a single exposure of the sample is required per projection. A low radiation dose is of particular value for in vivo phase contrast imaging of biological samples. The technique gives good phase sensitivity as a result of the sub-pixel accuracy of the DIC algorithms for measuring the speckle displacement. The distance between the membrane and the detector can be increased to several meters provided the beam transverse coherence is large enough to magnify the speckle displacement, and thus improves the sensitivity to the phase shift. The method is also only moderately demanding in terms of beam stability as only a pair of short exposure images are required, and the normalized correlation criterion renders the process insensitive to illumination changes^28^. At present, the computation time for data analysis can seem long, however this could be reduced by improving the efficiency of the digital correlation algorithm or by writing the code in a compiled language such as C. A further development would be to increase the spatial resolution by scanning the membrane either in one or two dimensions^23^,^24^. This processing would however require a much larger number of exposures and is then less attractive for dose sensitive samples. Another possible development would be application of the technique at higher X-ray energies replacing the phase object with a scattering object made of a heavier material. Preliminary test have shown that the speckle tracking could be efficient from 50 keV to 100 keV. Finally, XST based phase contrast imaging has been recently demonstrated using lab-based sources^31^, increasing the potential for applications in biology, medicine, non-destructive testing, food inspection and security.

## Methods

The initial experiment was performed on the Test beamline B16 at Diamond Light Source, and the data presented in this study were collected at the PETRA III beamline P05. A monochromatic beam produced by a double crystal monochromator (ΔE/E ≈ 10^−4^) at an X-ray energy of 15 keV and cut down to a cross section of 6.2 mm × 3.4 mm was calculated to give an X-ray flux of 4.5 × 10^13^ photons/mm^2^/sec. The random phase object was a sheet of silicon carbide abrasive paper (FEPA Grit P3000 with average particle diameter of 6.5 μm) which was mounted in the X-ray beam at a distance of 87.5 m from the source and close to the sample. The distance between the membrane and the detector was set to the maximum mechanically achievable length of 0.89 m in order to increase the sensitivity. The speckle pattern was recorded with a high-resolution imaging X-ray microscope with effective pixel size of 2.5 × 2.5 μm^2^. This imaging system consisted of a CdWO_4_ scintillator (250 μm thick) producing a visible-light image of the X-ray beam, a microscope objective, a mirror reflecting at 90°, and a PCO 4000 CCD camera with the physical pixel size of 12 μm × 12 μm and the exposure time was 1.2 seconds for each sample orientation. The sample was mounted on a vertical axis rotation stage and measurements were taken at angles from 0° to 180° in steps of 0.2°. After each 30° of rotation, 20 reference images of the membrane speckle with no sample present were taken to minimize the effect from beam instability due to the double crystal monochromator cooling system.

### Sample preparation

The specimen was provided by the Institute of Clinical Radiology, Ludwig-Maximilians-University Hospital (Munich, Germany) in accordance with the Declaration of Helsinki and approved by the local ethics committee. The artery was scanned in a plastic Eppendorf tube (10 mm diameter) fixated in a 4% buffered formaldehyde solution. A metal stent was used to mark the region of interest of the artery. The measurement was focused on the vessel part mainly consisting of fatty tissue.

### Reconstruction

Data processing was performed using a program run in Matlab version 2012b. The subset used in our analysis was of 11 × 11 pixels in size which gives a good compromise between accuracy and spatial resolution. Due to rapidly changing phase at the edges of the sample container, a mask was applied to the projected phase image to minimize the reconstructed phase error. Here, the edges of the sample container are defined as the mask boundary, and the phase outside of the mask boundary is set to zero. The compiled Matlab code was run using parallel processing distributed over 30 nodes of the Diamond Light Source computation cluster, taking 66 minutes to process all of the 900 datasets. Tomographic reconstruction was then performed on absorption and phase-shift images which were downsampled by 3, using 8 GPU accelerated computation nodes. The reconstruction algorithm was of the parallel-beam filtered-back-projection kind with GPU acceleration implemented in the CUDA programming language (Nvidia, USA) for speed. Ring artefact suppression was applied with the algorithm of Titarenko et al^32^.

## Author Contributions

H.W., K.S., S.B. and J.H. planned and designed the experiment. J.H., H.W. and A.H. conducted the experiment. H.W. and S.B. did the data analysis. R.A. and H.W. performed the tomographic reconstruction. H.W., D.L., S.B. and K.S. wrote the manuscript with inputs from all authors.
